# The *Non-Coding RNA* Journal Club: Highlights on Recent Papers—2

**DOI:** 10.3390/ncrna1020167

**Published:** 2015-09-22

**Authors:** Claire Francastel, Florent Hubé, Sendurai A. Mani, Gaetano Santulli, Joseph H. Taube, Zofia Szweykowska-Kulinska

**Affiliations:** 1 CNRS UMR7216, Epigenetics and Cell Fate, Université Paris Diderot, Sorbonne Paris Cité, UMR7216 Epigénétique et Destin Cellulaire, Bâtiment Lamarck B, Case Courrier 7042, 35 rue Hélène Brion, 75013 Paris, France; E-Mails: claire.francastel@univ-paris-diderot.fr (C.F.); florent.hube@univ-paris-diderot.fr (F.H.); 2 Department of Molecular Pathology, The University of Texas MD Anderson Cancer Center, Houston, TX 77054, USA; E-Mail: smani@mdanderson.org; 3 Department of Physiology and Cellular Biophysics, Clyde and Helen Wu Center for Molecular Cardiology, College of Physicians and Surgeons, Columbia University Medical Center, New York, NY 10032, USA; E-Mail: gs2620@cumc.columbia.edu; 4 Department of Biology, Baylor University, Waco, TX 76706, USA; E-Mail: jtaube@mdanderson.org; 5 Department of Gene Expression, Faculty of Biology, Adam Mickiewicz University, Umultowska 89, 61-614 Poznan, Poland

## 1. Introduction

We are glad to share with you our second *Journal Club* and to highlight some of the most interesting papers published recently. We hope we will tease your curiosity and encourage you to read full papers outside of your research area, which you may not have read otherwise.

The *Non-Coding RNA* Scientific Board wishes you an exciting read.

## 2. Insulin Receptor Substrate-1 Encodes Bifunctional RNAs

Highlight by Claire Francastel and Florent Hubé

Once again, bifunctional RNAs are in the spotlight with, this time, the work of Nagano *et al.* [1] who identified two main transcript isoforms for the Insulin receptor substrate-1 (Irs-1) gene, which differ in their 5′- and 3′-untranslated regions (UTR). Although both RNAs retain the coding capacity, the longest mRNA isoform (FL-Irs-1) functions as an inhibitor of Rb mRNA expression through a complementary sequence element present in the 5′-UTR, independently of the protein IRS-1. These data add an unconventional player to the long list of myogenic differentiation regulators and put a little more forward the non-coding functions that can hide within mRNA.

## 3. Polarity-Dependent microRNA Processing to Suppress Epithelial Mesenchymal Transition (EMT)

Highlight by Joseph H. Taube and Sendurai A. Mani

It was the best of times, it was the worst of times, for microRNA processing. At either end of an epithelial cell sits two different E-cadherin dependent complexes. While p120 interacts with E-cadherin in both the apical and basal compartment, PLEKHA7 is specific to the apical zonula adherens (ZA). Kourtidis *et al.* use proteomic and knockdown methods to show that the PLEKHA7, at the apical complex, is essential for suppressing anchorage-independent growth and expression of transformation-related markers, whereas knockdown of both PLEKHA7 and p120 rescued this effect, revealing an opposing function for the basal complex [2]. This is, in part, due to the recruitment of the microprocessor complex proteins DROSHA and DGCR8 outside of the nucleus and to the ZA. There, microprocessor facilitates the conversion of primary miR-30b (pri-miR-30b) to precursor miR-30b (pre-miR-30b), a process widely considered to occur exclusively in the nucleus. Among other targets, miR-30b is known to down-regulate expression of the epithelial-mesenchymal transition-inducing factor SNAI1, thus revealing an intracellular regulatory pathway that reinforces the coherence of epithelial surfaces.

## 4. Mitochondrial Calcium Overload and Oxidative Stress in Cardiomyocytes Are Linked by microRNA-25

Highlight by Gaetano Santulli

Chen’s group has elegantly demonstrated that miR-25 plays a mechanistic role in regulating mitochondrial function in cardiomyocytes [3]. The mitochondrial calcium uniporter (MCU), a critical Ca^2+^ transporter that regulates mitochondrial [Ca^2+^], was identified as a target of miR-25 by bioinformatic analysis. Intriguingly, miR-25 significantly decreased H_2_O_2_-induced elevation of mitochondrial Ca^2+^ concentration and protected cardiomyocytes against oxidative damage by inactivating the mitochondrial apoptosis pathway.

This discovery has major implications since mitochondrial Ca^2+^ overload has been recently determined as a key player in the pathophysiology of heart failure. Chen’s work establishes miR-25 as a specific regulator of MCU, providing fundamental bases towards the design of therapies that can regulate mitochondrial [Ca^2+^]. Indeed, while Ca^2+^ is needed in such organelle to activate some enzymes in the Krebs cycle, mitochondrial Ca^2+^ overload can be extremely detrimental.

## 5. Double Function of Elongator in miRNA Biogenesis

Highlight by Zofia Szweykowska-Kulinska

In their recent paper published in *Nature Plants*, Fang *et al.* [4] have shown the double role of Elongator in the case of plant miRNA biogenesis. Elongator complex, which plays versatile roles in transcription and RNA processing, is also involved in *MIR* genes transcription stimulation and pri-miRNA processing. Experiments have shown that Elongator interacts with Dicer-Like 1 (DCL1) containing Dicing complex and its disruption impairs DCL1 localization in nuclear Dicing bodies. Moreover, the association of DCL1 with chromatin is dependent on the presence of Elongator. Earlier experiments already suggested a negative correlation between DCL1 activity and among the other Arabidopsis pri-miRNA 163 splicing efficiency. A picture also arises showing multifunctional character of the DCL1 protein during miRNA biogenesis.
